# Mutations of tyrosine 467 in the human norepinephrine transporter attenuate HIV-1 Tat-induced inhibition of dopamine transport while retaining physiological function

**DOI:** 10.1371/journal.pone.0275182

**Published:** 2022-09-28

**Authors:** Matthew J. Strauss, Katherine D. Porter, Pamela M. Quizon, Sarah E. Davis, Steven Lin, Yaxia Yuan, Gustavo A. Martinez-Muniz, Wei-Lun Sun, Chang-Guo Zhan, Jun Zhu

**Affiliations:** 1 Department of Drug Discovery and Biomedical Sciences, College of Pharmacy, University of South Carolina, Columbia, SC, United States of America; 2 Molecular Modeling and Biopharmaceutical Center, University of Kentucky, Lexington, KY, United States of America; 3 Department of Pharmaceutical Sciences, College of Pharmacy, University of Kentucky, Lexington, KY, United States of America; 4 Department of Psychological Science, University of North Georgia, Dahlonega, GA, United States of America; Shanxi University, CHINA

## Abstract

Dysregulation of dopaminergic transmission induced by the HIV-1 transactivator of transcription (Tat) has been implicated as a central factor in the development of HIV-1 associated neurocognitive disorders (HAND). We have demonstrated that the tyrosine470 residue of the human dopamine transporter (hDAT) plays a critical role in Tat-hDAT interaction. Based on the computational modeling predictions, the present study sought to examine the mutational effects of the tyrosine467 residue of the human norepinephrine transporter (hNET), a corresponding residue of the hDAT tyrosine470, on Tat-induced inhibition of reuptake of dopamine through the hNET. Mutations of the hNET tyrosine467 to a histidine (Y467H) or a phenylalanine (Y467F) displayed similar kinetic properties of reuptake of [^3^H]dopamine and [^3^H]norepinephrine in PC12 cells expressing wild-type hNET and its mutants. Compared to wild-type hNET, neither of Y467H or Y467F altered B_max_ and K_d_ values of [^3^H]WIN35,428 binding, whereas Y467H but not Y467F decreased the B_max_ of [^3^H]nisoxetine binding without changes in K_d_. Y467H also increased the affinity of nisoxetine for inhibiting [^3^H]dopamine uptake relative to wild-type hNET. Recombinant Tat_1-86_ (140 nM) induced a significant reduction of [^3^H]dopamine uptake in wild-type hNET, which was attenuated in both Y467H and Y467F. Compared to wild-type hNET, neither Y467H or Y467F altered [^3^H]dopamine efflux in CHO cells expressing WT hNET and mutants, whereas Y467F but not Y467H decreased [^3^H]MPP^+^ efflux. These results demonstrate tyrosine467 as a functional recognition residue in the hNET for Tat-induced inhibition of dopamine transport and provide a novel insight into the molecular basis for developing selective compounds that target Tat-NET interactions in the context of HAND.

## Introduction

Despite the success of combinatorial antiretroviral therapy (cART) in controlling peripheral HIV infection and improving the lives of HIV patients, an estimated 50% of the 38 million HIV-positive patients have neurophysiological dysfunction and HIV-associated neurocognitive disorders (HAND) [1, 2]. Most cART medications cannot cross the blood-brain barrier, while infected macrophages carrying the virus can [3], allowing the CNS to serve as an HIV-viral reservoir [4, 5]. The replication and expression of viral proteins in the CNS are associated with the persistence of HIV-related neuropathology and subsequent neurocognitive deficits [4, 6–8] and are central to the development of HAND [9]. Among the viral proteins, the transactivator of transcription (Tat) protein plays a crucial role in the neurotoxicity and cognitive impairment evident in HAND [10, 11]. The HIV-1 Tat protein is a major pathogenic factor for HAND. The Tat protein is secreted from HIV-infected microglia and astrocytes then taken up by neurons, leading to neuronal damages [4, 12–16]. The Tat can be detected in the human cerebrospinal fluid (CSF) of HIV-positive individuals on suppressive cART with low plasma viral load [17–20] and in the human brain of HIV-infected individuals who are cART naïve [21]. The detectable Tat in human specimen of aviremic patients demonstrates a causal effect of HIV-associated cognitive impairment [17, 22]. The Tat protein induces neurophysiological and anatomical deficits observed in neuroHIV [23–26], including bystander neuronal injury and loss of synaptic connectivity [27–29]. Considering that long-term viral protein exposure can accelerate damage to the mesocorticolimbic dopamine (DA) system [30–32], defining the molecular mechanism(s) by which the HIV-1 Tat impairs the DA system and affects the progression of HAND may provide valuable insight into the development of novel therapies for this population.

Clinical observations revealed that DA levels are increased in the CSF of therapy naïve HIV patients in asymptomatic infection [33] and HIV-infected individuals on active cART [34]. However, the decreased CSF DA levels are found in postmortem brains [35–37] and CSF in HIV positive individuals in both the pre-cART [38, 39] and the post-cART groups [40, 41]. The potential effect of the decreased CSF Tat levels on the DA system has translated in HIV infected patients on cART with parkinsonism [42–46]. Dopamine (DA) transporter (DAT) transports the extracellular DA into cytosolic space of the synaptic terminals, whereas the vesicular monoamine transporter2 transports the cytosolic DA into synaptic vesicles, where both the DAT and vesicular monoamine transporter2 are critical for normal DA homeostasis. Our published work has demonstrated that HIV-1 Tat protein decreases DA transport through DAT in cells expressing human DAT [47–49] and in the prefrontal cortex of inducible Tat transgenic (iTat-tg) mice [50]. Importantly, HIV-induced elevated DA levels in CNS can stimulate viral replication in human macrophages within DA-rich brain regions [9, 51, 52], resulting in viral protein release, which has been implicated in the pathophysiology of HAND [53].

Monoamine transporters are responsible for the reuptake of their respective neurotransmitters by DAT, NE transporter (NET), and serotonin transporter [54]. The prefrontal cortex is a critical brain region for higher cognitive function [55–57]. Both DAT and NET in the prefrontal cortex can transport DA, whereas the serotonin transporter has too weak an affinity to effectively take up and transport DA at physiological levels [58, 59]. Our recent studies demonstrated that DA uptake via DAT and NET is decreased in the prefrontal cortex of inducible Tat_1-86_ transgenic mice [50]. Therefore, determining the mechanistic basis underlying the Tat interactions with DAT and NET may reveal novel therapeutic possibilities for preventing the increase in comorbid conditions as well as HAND. Through a combined computational modeling and experimental validation approach, we demonstrated that DAT tyrosine470 (Tyr470) is one of the key residues involved in Tat and DAT interaction and mutation of this residue (Tyr470 to a histidine, Y470H) attenuates Tat-induced inhibition of DA uptake in cells expressing wild type hDAT and mutant [47, 48]. The integrated computational modeling and experimental validation study also demonstrated interaction of hNET with HIV-1 Tat [60]. The current study further explored the role of tyrosine467 (Tyr467) in the hNET-Tat binding. Based on molecular modeling, the role of Tyr467 of hNET in the hNET-Tat binding should be similar to that of Tyr470 of hDAT in the hDAT-Tat binding [61]. Therefore, we determined the mutational effect of Tyr467 on basal transport function of hNET and Tat-induced inhibition of hNET transport function by mutating Tyr467 to either a histidine (Y467H) or a phenylalanine (Y467F). The obtained experimental data confirmed the role of Tyr467 of hNET in the hNET-Tat binding.

## Materials and methods

### Materials

[^3^H]DA (3,4-ethyl-2[N-P^3^PH]dihydroxyphenylethylamine; specific activity, 40 Ci/mmol) and [^3^H] norepinephrine (specific activity 14.5 Ci/mmol) were purchased from American Radiolabeled Chemicals, Inc (St. Louis, MO). [^3^H]WIN 35,428 (specific activity, 85 Ci/mmol), [^3^H]Nisoxetine (specific activity 82.0 Ci/mmol), and [^3^H]MPP^+^ (82.9 Ci/mmol) were purchased from PerkinElmer Life and Analytical Sciences (Boston, MA). Recombinant HIV-1 transactivator of transcription (rTat_1–86_) protein was purchased from ImmunoDX (Woburn, MA). Antibodies recognizing anti-hNET (NET17-1, mouse monoclonal antibody), anti-mouse IgG horseradish peroxidase (anti-mouse-HRP, #7076), and anti-Calnexin (sc-11397; rabbit polyclonal antibody) were purchased from Mab Technology (Neenah, WI), Cell Signaling Technology (Danvers, MA) and Santa Cruz Biotechnology, Inc. (Dallas, TX), respectively. PC12 cells (ATCC^®^ CRL-1721.1), Chinese hamster ovarian cells (CHO-K1, ATCC^®^ CCL-61), and RPMI-1640 and F-12K cell medium were purchased from American Type Culture Collection (Manassas, VA). Fetal bovine serum was purchased from Atlanta Biologicals (Flowery Branch, GA). Horse serum, penicillin/streptomycin, and trypsin/EDTA were purchased from Fisher Scientific (Waltham, MA). D-Glucose, L-ascorbic acid, cocaine, nisoxetine, WIN35,428, nomifensine, desipramine, DMSO, bovine serum albumin, pyrocatechol, α-D-glucose, HEPES, isopropanol, sucrose, 2- and Tween 20 were purchased from Sigma-Aldrich (St. Louis, MO).

#### Molecular modeling of hNET binding with HIV-1 Tat

The complex structure of hNET binding with HIV-1 Tat was constructed based on our previous reported hDAT-Tat complex structure [62] via homology modeling and molecular dynamics (MD) simulation [60]. The last snapshot of the MD-simulated hNET-Tat binding complex structure was energy-minimized for the binding analysis.

#### Construction of plasmids

**Fig 1:**
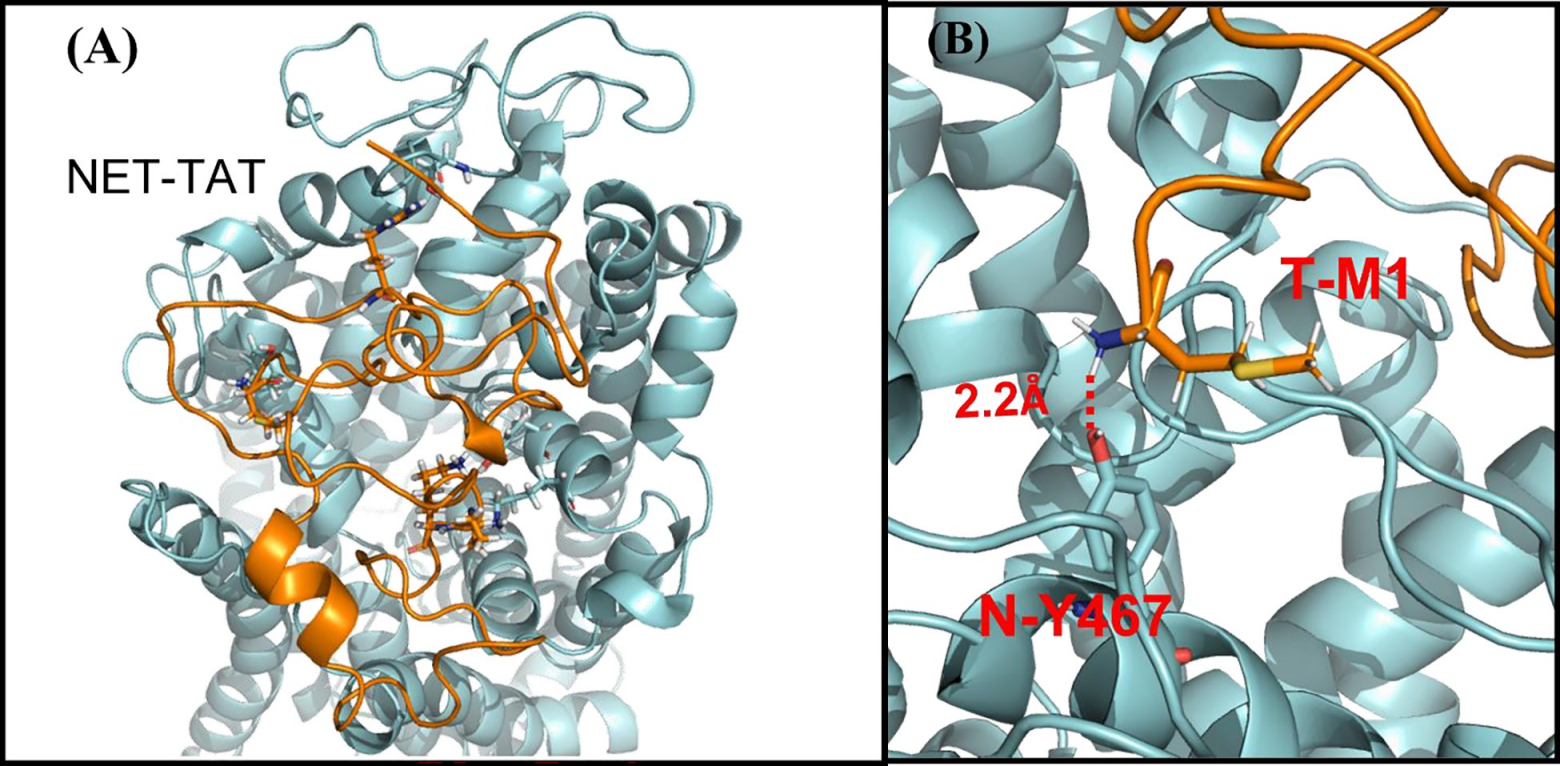
Overview of hNET-Tat complex structure. (A) hNET and Tat are showed in cyan and gold surface style, respectively. (B) Local view of the favorable hydrogen bond between the positively charged N-terminal amino group of residue M1 in Tat and the hydroxyl group of residue Y467 in hNET with labeled distance.

The Tyr467 residue on hNET was chosen based on predictions of computational modeling and simulations (Fig 1) and our previous studies showing Tyr470, a homologous residue on hDAT [47, 48, 61, 62]. Mutations of Tyr467 to histidine (Y467H) or phenylalanine (Y467F) are expected to abolish a critical hydrogen bond between hNET and HIV-1 Tat. Y467H andY467F were generated based on the wild type hNET (WT hNET) sequence (NCBI, cDNA clone MGC: 190603 IMAGE: 100062757) by site-directed mutagenesis. Synthetic cDNA encoding hNET subcloned into pcDNA3.1+ was used as a template to generate mutants using the QuikChange™ site-directed mutagenesis Kit (Agilent Tech, Santa Clara CA). The sequences of the mutant constructs were confirmed at the University of South Carolina EnGenCore facility. DNA plasmids were propagated and purified using a Qiagen Plasmid Maxi Kit (Valencia, CA).

#### Cell culture and DNA transfection

All cell cultures and transfection were conducted as described previously [47]. PC12 cells were maintained at 37°C in a 5% CO_2_ incubator in Dulbecco’s modified eagle medium (DMEM, Life Technologies, Carlsbad, CA) supplemented with 15% horse serum, 2.5% fetal bovine serum, 2 mM glutamine, and antibiotics (100 U/ml penicillin and 100 μg/mL streptomycin). CHO cells were maintained at 37°C in a 5% CO_2_ incubator with medium supplemented with 11% fetal bovine serum and 5% antibiotics (100 U/ml penicillin and 100 μg/mL streptomycin). For the intact cell experiments, once cells grew to 80–90% confluence on 10 cm plates they were then seeded at a density of 1 × 10^5^ cells/cm^2^ in 24-well plates. Precoated 24-well plates with poly-D-lysine were purchased from Corning, Inc. (Corning, NY). Twenty-four hours after seeding, cells were transfected with 0.8 μg of WT hNET, Y467H, or Y467F plasmid DNA per well using Lipofectamine 2000 (Life Technologies). Cells were used for experiments 24 h after transfection.

#### Kinetic analysis of [^3^H]dopamine and [^3^H]norepinephrine uptake assays

The maximal velocity (V_max_) and Michaelis-Menton constant (K_m_) of [^3^H]Dopamine ([^3^H]DA) or [^3^H]Norepinephrine ([^3^H]NE) uptake was evaluated in PC12 cells transfected with WT hNET, Y467H, or Y467F using a procedure modified from our previous reports [47, 49]. Twenty-four hours following transfection, intact PC12 cells in 24-well plates were washed twice with Krebs-Ringer-HEPES (KRH) buffer (final concentration in mM: 125 NaCl, 5 KCl, 1.5 MgSO_4_, 1.25 CaCl_2_, 1.5 KH_2_PO_4_, 10 D-glucose, 25 HEPES, 0.1 EDTA, 0.1 pargyline, and 0.1 L-ascorbic acid; pH 7.4). Cells were then preincubated for 10 min at room temperature in 450 μL of KRH buffer with or without 10 μM nomifensine (for [^3^H]DA uptake) or 10 μM desipramine (for [^3^H]NE uptake) to determine nonspecific binding. Following the 10 min incubation, cells were incubated for an additional 8 min at room temperature in the presence of one of six concentrations of unlabeled DA or NE (final concentrations 0.03–5 μM) combined with a fixed concentration of [^3^H]DA (500,000 dpm/well) or [^3^H]NE (500,000 dpm/well), respectively. Specific hNET-mediated DA or NE uptake was calculated by subtracting the non-specific uptake (in the presence of nomifensine or desipramine) from the total uptake. The reaction was terminated by the removal of solution from the wells followed quickly by three washes with ice-cold KRH buffer. Cells were then lysed in 500 μL of 1% SDS for one hour and radioactivity was measured using a liquid scintillation counter (Tri-Carb 2900TR; PerkinElmer Life and Analytical Sciences, Waltham, MA).

To determine whether Y467H or Y467F alter the affinity of NET substrates or inhibitors, the competitive inhibition of DA or NE uptake via hNET was determined in intact PC12 cells transfected with WT hNET or mutants as described above. Cells were preincubated with a series of final concentrations of DA (1 nM– 1 mM), nisoxitine (0.1 nM– 100 μM), or cocaine (1 nM– 100 μM) at room temperature for 10 min followed by an additional 8 min incubation with a fixed concentration of [^3^H]DA (50 nM, final concentration). To assess whether the Y467H or Y467F mutations could attenuate the inhibitory effects of HIV-1 Tat on hNET, we performed [^3^H]DA uptake in PC12 cells transfected with WT hNET or mutants in the presence or absence of HIV-1 Tat protein. Cells were detached from 10 cm culture dishes with trypsin/EDTA (0.25%/0.1%), resuspended in culture medium, and incubated at room temperature for 10 min. The cell suspensions were pelleted by centrifugation at 400 × g for 5 min at 4°C and then washed once with phosphate-buffered saline followed by additional 5 min centrifugation (400 × g, 4°C). The resulting cell pellets were then resuspended in KRH assay buffer. The cell suspensions from WT hNET, Y467H, or Y467F mutants were then preincubated with or without rTat_1–86_ (140 nM, final concentration) at room temperature for 20 min followed by additional 8 min incubation with mixed [^3^H]DA (50 nM, final concentration) as reported previously [47, 49]. Non-specific [^3^H]DA uptake was determined in the presence of 10 μM desipramine. The reaction was terminated by immediate filtration through Whatman GF/B glass filters (presoaked for 2 h with 1 mM pyrocatechol) followed by three washes with ice-cold KRH buffer containing pyrocatechol using a Brandel cell harvester (model M-48; Brandel Inc., Gaithersburg, MD). Radioactivity was determined as described above.

#### [^3^H]WIN 35,428 and [^3^H]nisoxetine binding assays

[^3^H]WIN 35,428 and [^3^H]Nisoxetine represent binding sites on hNET with different kinetic profiles [63]. To determine whether mutation of Tyr467 alters hNET binding sites, we determined the kinetic parameters (B_max_ and K_d_) of [^3^H]WIN 35,428 or [^3^H]Nisoxetine in PC12 cells transfected with WT hNET or mutants. Cell suspensions were prepared as described above. Following cell suspension preparation, the final cell pellets were resuspended in 1 mL of assay buffer (15 mM Na_2_HPO_4_, 30 mM NaH_2_PO_4_, 122 mM NaCl, 5 mM KCl, 1 mM MgSO_4_, 10 mM glucose, 1 mM CaCl, and 10 nM EDTA). For the [^3^H]WIN 35,428 binding assay, 25 μL of cell suspension was added to tubes containing 200 μL of assay buffer with or without 10 μM desipramine (for nonspecific binding) and preincubated for 5 min at room temperature. Following the preincubation, 25 μL of one of six concentrations of unlabeled WIN35,428 (final concentrations 1–30 nM) combined with a fixed concentration of [^3^H]WIN 35,428 (250,000 dpm/well) was added to each tube (final volume 250 μL) and incubated for an additional 15 min at room temperature. The reaction was terminated by immediate filtration through Whatman GF/B glass filters (presoaked for 2 h with 1 mM pyrocatechol) followed by three washes with ice-cold assay buffer using a Brandel cell harvester (model M-48; Brandel Inc., Gaithersburg, MD) and radioactivity was determined as described above. The [^3^H]Nisoxetine binding assays were performed identically to the [^3^H]WIN 35,428 binding assays with the following modifications: (1) The same concentrations of unlabeled and [^3^H]Nisoxetine (250,000 dpm/well) were used instead of [^3^H]WIN 35,428, and (2) instead of being resuspended in 1 mL assay buffer, for the [^3^H]Nisoxetine binding assay, the cells were counted after being suspended in 1 mL assay buffer, and then diluted to a concentration of 100,000 cells/ 25 μL.

#### Cell surface biotinylation and western blots

To determine whether the hNET mutations redistribute NET in presynaptic plasma membranes, biotinylation assays were performed as described previously [49]. PC12 cells were cultured on 6-well plates at a density of 10^5^ cells/well. 24 hours after seeding in the 6-well plates, PC12 cells were transfected with WT hNET, Y467H, or Y467F hNET as described above. Cells were incubated with 1 mL of 1.5 mg/mL of sulfo-NHS-SS biotin (Pierce, Rockford, IL) in PBS/Ca/Mg buffer (138 mM NaCl, 2.7 mM KCl, 1.5 mM KH_2_PO_4_, 9.6 mM Na_2_HPO_4_, 1 mM MgCl_2_, and 100 nM CaCl_2_, pH 7.3). After incubation, cells were washed 3 times with 1 mL of ice-cold PBS/Ca/Mg buffer containing 100 mM glycine and incubated for 30 min at 4°C in the same buffer. Cells were then washed 3 times with 1 mL of ice-cold PBS/Ca/Mg buffer and then lysed by addition of 500 ml of Lysis buffer (1% Triton X-100, 10 mM Tris HCl, 150 mM NaCl, 1 mM EDTA, and 250 μM phenylmethanesulfonyl fluoride), followed by incubation and continuous shaking for 20 min at 4°C. Cells were transferred to 1.5 mL tubes and centrifuged at 20,000 × g for 20 min. The resulting pellets were discarded, and 100 μL of the supernatants were stored at -20°C for determination of total NET. Remaining supernatants were incubated with continuous shaking in the presence of monomeric avidin beads in Triton X-100 buffer (100 μL/tube) for 1 h at room temperature. Samples were centrifuged subsequently at 17,000 × g for 4 min at 4°C, and supernatants (containing the non-biotinylated, intracellular protein fraction) were stored at -20°C. Resulting pellets containing the avidin-absorbed biotinylated proteins (cell-surface fraction) were resuspended in 1 mL of 1.0% Triton X-100 buffer and centrifuged at 17,000 × g for 4 min at 4°C, and pellets were resuspended and centrifuged twice. Final pellets consisted of the biotinylated proteins adsorbed to monomeric avidin beads. Biotinylated proteins were eluted by incubating with 50 μL of Laemmli buffer for 20 min at room temperature and stored at -20°C for western blotting.

To obtain immunoreactive NET protein in total synaptosomal, intracellular, and cell surface fractions, samples from different fractions were thawed and subjected to gel electrophoresis and western blotting. Samples were separated by 10% SDS-polyacrylamide gel electrophoresis for ~90 min at 125V. Samples were then transferred to Immobilon-P transfer membranes (0.45 μm pore size; Millipore Co., Bedford MA) in transfer buffer (50 mM Tris, 250 mM glycine, 3.5 mM SDS) using a Mini Trans-Blot Electrophoretic Transfer Cell (Bio-Rad; Hercules, CA) for 90 min at 75 V. The membranes were then incubated with blocking buffer (5% milk powder in PBS containing 0.5% Tween-20) for 1 h at room temperature, followed by incubation with mouse anti-NET (diluted 1:1,000 in blocking buffer) overnight at 4°C. Transfer membranes were then washed three times with blocking buffer at room temperature followed by incubation with anti-mouse-HRP (diluted 1:5,000 in blocking buffer) for 1 h at room temperature. Membranes were then washed an additional three times in PBS containing 0.5% Tween-20 (Sigma-Aldrich; St. Louis, MO). Immunoreactive proteins on the transfer membranes were detected using Amersham ECL prime western blotting detection reagent (GE life sciences; Chicago, IL) and developed on Hyperfilm (GE life sciences; Chicago, IL). After detection and quantification of NET each blot was washed and re-probed with anti-Calnexin antibody (diluted 1:10,000 in blocking buffer), an endoplasmic reticular protein, to monitor protein loading between all groups. Multiple autoradiographs were obtained using different exposure times, and immunoreactive bands within the linear range of detection were quantified by densitometric scanning using Scion image software. Band density measurements, expressed as relative optical density, were used to determine levels of NET in the total synaptosomal fraction, the intracellular fraction (non-biotinylated), and the cell surface fraction (biotinylated).

#### [^3^H]dopamine and [^3^H]MPP^+^ efflux assays

To determine whether the mutated Y467 in hNET alters transporter conformational transients, DA or MPP^+^ efflux was performed as described previously [48]. Intact CHO cells in precoated Poly-D-Lysine 24-well plates (Ref# 356414, Corning, Kennebunk, ME) were transfected with WT hNET or its mutants and preloaded with [^3^H]DA (50 nM final concentration) for 20 minutes or [^3^H]MPP^+^ (5 nM final concentration) for 30 minutes at room temperature. After the incubation period, cells were then washed 2 times with 1 × KRH buffer before collecting fractional release samples. Total uptake of [^3^H]DA or [^3^H]MPP^+^ at zero time point was estimated from four wells per genotype that were lysed immediately with 1% SDS after the incubation period. Nonspecific uptake of [^3^H]DA or [^3^H]MPP^+^ at zero time point was estimated from four wells per genotype that were incubated with the hNET inhibitors desipramine and nomifensine (100 μM and 10 μM final concentration, respectively) and lysed immediately with 1% SDS after the incubation period. To collect fractional release, 500 μL of KRH buffer was added into a set of four wells and transferred to scintillation vials after 0 minutes as the initial fractional efflux. Fresh 500 μL of KRH buffer was added into the wells and incubated for 10 minutes then transferred to scintillation vials. Fractional efflux was collected every 10 minutes for 90 minutes ([^3^H]DA) or 110 minutes ([^3^H]MPP^+^). After the last fractional efflux was collected, the cells were lysed and samples collected as leftover [^3^H]DA or [^3^H]MPP^+^ remaining in the cells.

#### Statistical analysis

Results are presented as mean ± SEM, and *n* represents the number of independent experiments for each experiment group. Kinetic parameters (V_max,_ K_m_, B_max_, and K_d_) were determined from saturation curves by nonlinear regression analysis using a one-site model with variable slope. IC_50_ values for substrate and inhibitors for [^3^H]DA or [^3^H]NE uptake were determined from inhibition curves by nonlinear regression analysis using a one-site model with variable slope. For experiments involving comparisons between unpaired samples, unpaired Student’s *t* tests were used to assess any difference in the kinetic parameters (V_max_, K_m_, B_max_, K_d_ or IC_50_) between WT and hNET mutants; log-transformed values of IC_50_, K_m_ or K_d_ were used for the statistical comparisons. One way ANOVA with repeat measurement was used for analyzing the efflux data. Significant differences between samples were analyzed with separate ANOVAs followed by post-hoc tests, as indicated in the Results Section of each experiment. All statistical analyses were performed using IBM SPSS Statistics version 28, and differences were considered significant at *p* < 0.05.

## Results

### Computational modeling: Tyr467 and functional relevant residues of human NET

The computationally modeled hNET-Tat binding structure is depicted in Fig 1. According to the modeled binding structure, as shown in Fig 1B, the hydroxyl group of Y467 residue of hNET has a favorable hydrogen bond with the positively charged amine group of M1 residue of Tat. This data suggests that the Y467 residue is critical for HIV-1 Tat to bind to hNET, and subsequently inhibit its function. If this is true, mutation of Y467 to another residue with a non-aromatic side chain should considerably weaken the favorable hNET-Tat binding. In order to assess this hypothesis, we characterized the effects of mutating Y467 on hNET to either a Histidine (H) or a Phenylalanine (F) on physiologic transporter function and their ability to attenuate HIV-1 Tat-induced inhibition of hNET-mediated DA or NE uptake.

### Mutational effects of Tyr467 on hNET differentially influence DA or NE uptake kinetics

To determine the functional influence of Tyr467 mutations on hNET function, kinetic analysis of [^3^H]DA or [^3^H]NE uptake was performed in PC12 cells transfected with WT hNET, Y467A, or Y467F. As shown in Table 1, compared to WT hNET (V_max_: 57.0 ± 6.3 fmol/min/10^6^ cells, K_m_: 0.219 ± 0.063 μM), neither V_max_ or K_m_ of [^3^H]DA uptake was altered in Y467H (V_max_: 54.3 ± 6.7 fmol/min/10^6^ cells, K_m_: 0.225 ± 0.049 μM) and Y467F (V_max_: 59.3 ± 5.2 fmol/min/10^6^ cells, K_m_: 0.129 ± 0.017 μM). For [^3^H]NE uptake, no significant difference in V_max_ was observed among WT hNET (52.6 ± 15 fmol/min/10^6^), Y467H (74.4 ± 16 fmol/min/10^6^), and Y467F (86.2 ± 30 fmol/min/10^6^). Compared to WT hNET (0.179 ± 0.018 μM), K_m_ was not significantly altered in Y467H (0.597 ± 0.23 μM), but there was a 2-fold increase in Y467F (0.332 ± 0.026 μM, [*t*_(6)_ = 4.7; *p* < 0.01, unpaired Student’s *t* test]). This change suggests that mutating the Y to F mutation decreases the affinity of NE to its specific uptake site on hNET.

**Table 1:** Kinetic properties of the reuptake of [^3^H]DA and [^3^H]NE in PC12 cells expressing WT hNET and its mutants.

	WT hNET	Y467H	Y467F
[^3^H]DA uptake			
V_max_ (fmol/min/10^6^)	57.0 ± 6.3	54.3 ± 6.7	59.3 ± 5.2
K_m_ (μM)	0.219 ± 0.063	0.225 ± 0.049	0.129 ± 0.017
[^3^H]NE uptake			
V_max_ (fmol/min/10^6^)	52.6 ± 15	74.4 ± 16	86.2 ± 30
K_m_ (μM)	0.179 ± 0.018	0.597 ± 0.230	0.332 ± 0.026*

Data are presented as mean ± S.E.M. values from four independent experiments performed in duplicate.

* *p* < 0.05, unpaired student’s *t* test compared to WT hDAT.

**Fig 2:**
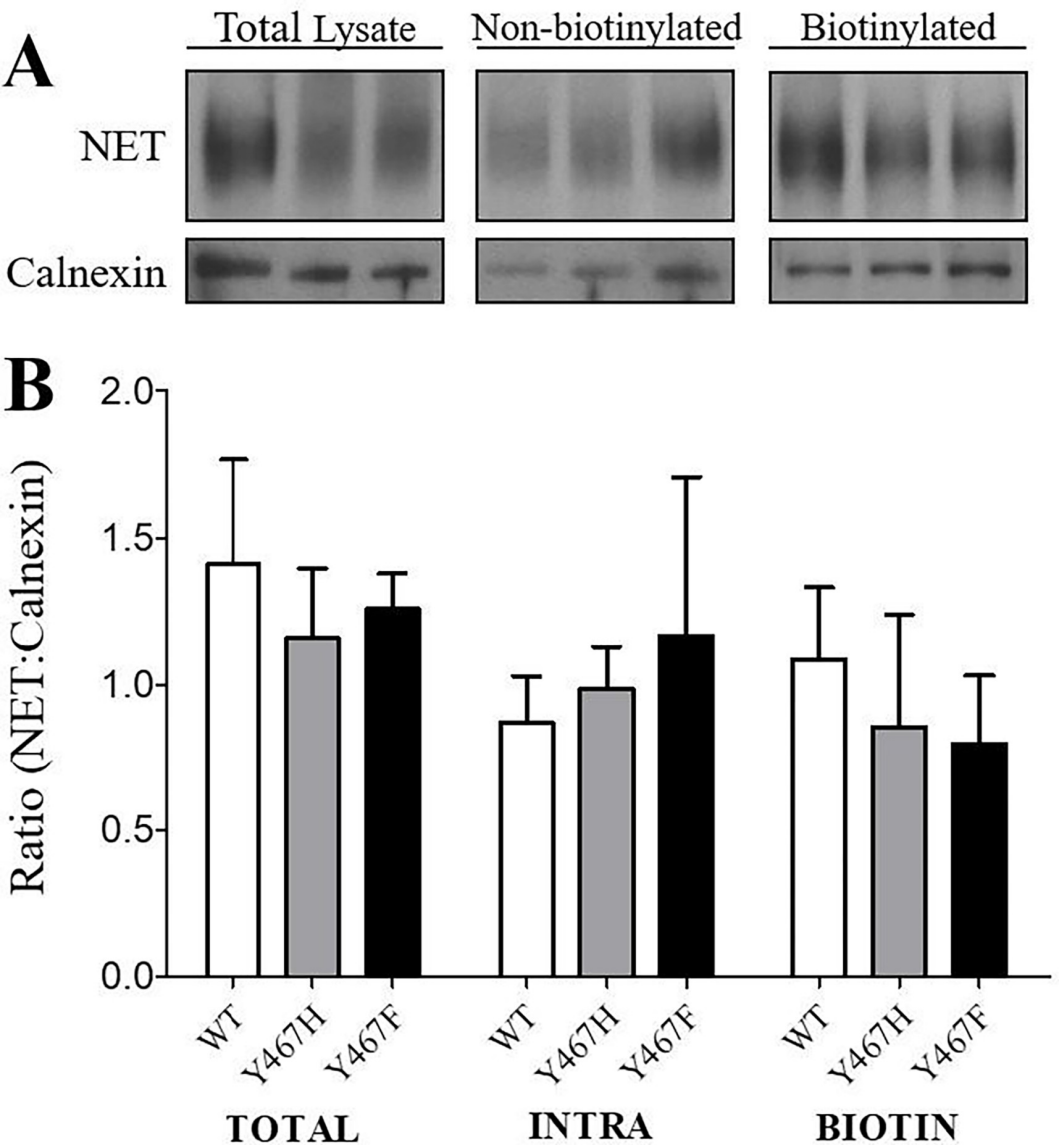
Cell surface expression of WThNET, Y467F, and Y467H was determined by biotinylation and Western blotting. (A) Representative immunoreactive blots for NET and calnexin (as control protein) in total, non-biotinylated (intracellular) and biotinylated (surface expression) fractions. (B) The quantification of total, non-biotinylated (INTRA) and biotinylated (BIOTIN) expression of NET was expressed as mean ± S.E.M of the ratio of total, non-biotinylated or biotinylated NET immunoreactivity to Calnexin immunoreactivity from 5 independent experiments. Raw immunoblots are provided in S1 File.

To determine whether mutations of Tyr467 alter subcellular distribution of NET, surface biotinylation followed by Western blot was performed. Three subcellular fractions were prepared from PC12 cells transfected with WT hNET, Y467H, and Y467F. NET immunoreactivity in total, intracellular (non-biotinylated), and the cell surface fraction (biotinylated) were examined (Fig 2). No differences between WT hNET and Y467H or Y467F were found in the ratio of total, nonbiotinylated or biotinylated NET to calnexin immunoreactivity, indicating that mutations of Tyr467 do not alter NET surface expression.

### Mutations of Tyr467 did not alter [^3^H]WIN 35,428 or [^3^H]Nisoxetine binding to NET

We next examined the B_max_ and K_d_ values of [^3^H]WIN 35,428 or [^3^H]Nisoxetine binding to WT hNET, Y467H, and Y467F. As shown in Table 2, compared to WT hNET (0.267 ± 0.063 pmol/10^6^ cells), the B_max_ values of [^3^H]WIN35,428 binding were not altered in Y467H (0.209 ± 0.034 pmol/10^6^ cells) or Y467F (0.322 ± 0.078 pmol/10^6^ cells). Compared to WT hNET (24.7 ± 3.1 nM), the K_d_ value was not altered in Y467H (31.0 ± 3.1 nM), but increased in Y467F [34.2 ± 1.9 nM, *t*_(6)_ = 2.5; *p* < 0.05, unpaired Student’s *t* test]. For [^3^H]Nisoxetine binding (Table 2), compared to WT hNET (0.261 ± 0.023 pmol/10^6^ cells), the B_max_ value was decreased in Y467H [0.183 ± 0.019 pmol/10^6^ cells, *t*_(9)_ = 2.5; *p* < 0.05, unpaired Student’s *t* test] but not altered in Y467F (0.273 ± 0.031 pmol/10^6^ cells). Additionally, no changes to the K_d_ value of [^3^H]Nisoxetine binding were found between WT hNET (8.86 ± 1.0 nM) and Y467H (9.44 ± 1.3 nM) or Y467F (9.61 ± 1.2 nM). Overall this data suggests that mutating Y467 to either Y467H or Y467F results in modest yet detectable alterations in transporter conformational states, which may affect the binding of substrates to the transporter.

**Table 2:** Kinetic properties of [^3^H] WIN35,428 and [^3^H] Nisoxetine binding in PC12 cells expressing WT hNET and its mutants.

	WT hNET	Y467H	Y467F
[^3^H]WIN 35,428			
B_max_ (pmol/10^6^)	0.267 ± 0.063	0.209 ± 0.034	0.322 ± 0.078
K_d_ (nM)	24.7 ± 3.1	31.0 ± 3.1	34.2 ± 1.9*
[^3^H]Nisoxetine			
B_max_ (pmol/10^6^)	0.261 ± 0.023	0.183 ± 0.019*	0.273 ± 0.031
K_d_ (nM)	8.86 ± 1.0	9.44 ± 1.3	9.61 ± 1.2

Data are presented as mean ± S.E.M. values from five to six independent experiments performed in duplicate.

* *p* < 0.05, unpaired student’s *t* test compared to WT hDAT.

### Mutational effects of Y467 on inhibition potency of DA or NE uptake by substrate and inhibitors

To further determine whether Tyr467 mutations influence selective binding sites of substrates or inhibitors on hNET, we determined the ability of substrate (DA or NE) and NET inhibitors (nisoxetine and cocaine) to inhibit [^3^H]DA or [^3^H]NE uptake in WT hNET and mutants (Table 3). Compared to WT hNET (DA, 174 ± 20 nM, cocaine, 4377 ± 904 nM), the apparent affinity (IC_50_) values for DA or cocaine inhibiting [^3^H]DA uptake were not altered in Y467H (DA, 236 ± 21 nM, Cocaine, 3920 ± 1010 nM), or Y467F (DA, 125 ± 12 nM, Cocaine, 4814 ± 964 nM). Compared to WT hNET (26.7 ± 4.0 nM), the IC_50_ for nisoxetine inhibiting [^3^H]DA uptake was significantly reduced in Y467H [15.9 ± 5.0 nM, *t*_(4)_ = 3.0; *p* < 0.05, unpaired Student’s *t* test] but not altered in Y467F (21.1 ± 5.1 nM). Compared to WT hNET (NE, 775 ± 218, nisoxetine, 10.1 ± 2.3 nM, and cocaine, 6959 ± 2130 nM), the IC_50_ values for NE, nisoxetine, and cocaine inhibiting [^3^H]NE uptake were not altered in Y467H (NE, 894 ± 258 Nisoxetine, 9.69 ± 4.7 nM, Cocaine, 2258 ± 899 nM) or Y467F (NE, 802 ± 106, Nisoxetine, 8.00 ± 3.4 nM, Cocaine, 2888 ± 1320 nM). The selective decrease in the IC_50_ of [^3^H]DA uptake, which was only observed in response to nisoxetine in the Y467H mutant, is in accordance with subtle differences between the nisoxetine and cocaine binding sites on hNET, and indicate that this mutation may specifically affect the nisoxetine site.

**Table 3:** Inhibitory affinity of substrates and inhibitors for [^3^H]DA and [^3^H]NE uptake in WT hNET and its mutants.

	WT hNET	Y467H	Y467F
IC_50_ for [^3^H]DA uptake (nM)			
DA	174 ± 20	236 ± 21	125 ± 12
Nisoxetine	26.7 ± 4.0	15.9 ± 5.0*	21.1 ± 5.1
Cocaine	4377 ± 904	3920 ± 1010	4814 ± 964
IC_50_ for [^3^H]NE uptake (nM)			
NE	775 ± 218	894 ± 258	802 ± 106
Nisoxetine	10.1 ± 2.3	9.69 ± 4.7	8.00 ± 3.4
Cocaine	6959 ± 2130	2258 ± 899	2888 ± 1320

Data are presented as mean ± S.E.M. values from five to six independent experiments performed in duplicate.

* *p* < 0.05, unpaired student’s *t* test compared to WT hDAT.

### Y467H and Y467F attenuate Tat-induced inhibition of DA transport

**Fig 3:**
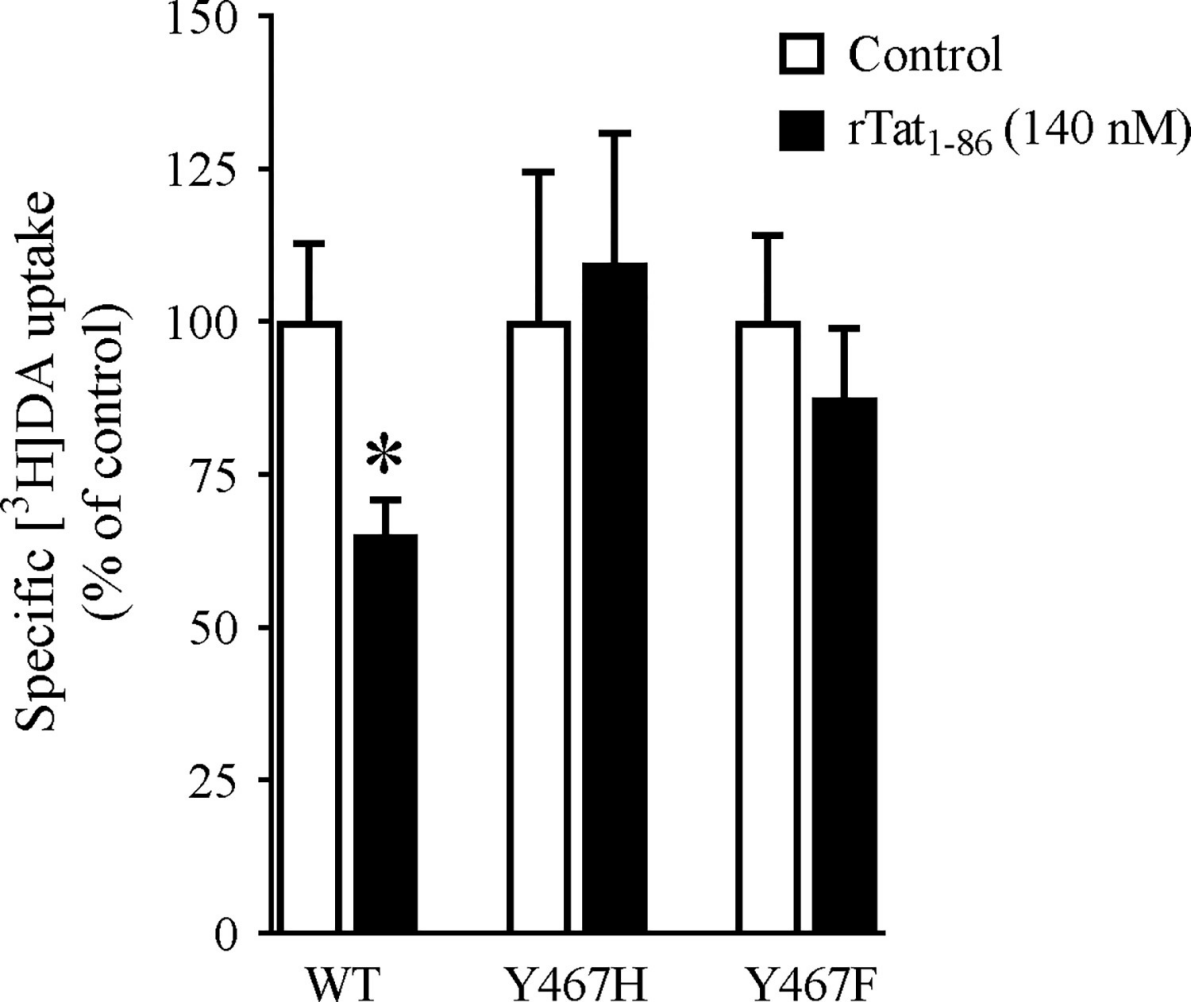
Effects of Tat_1-86_ on the specific [^3^H]DA uptake in WT-, Y467H-, and Y467F-hNET. PC12 cells transfected with the WT hNET or mutants were preincubated with or without recombinant Tat_1-86_ (rTat_1-86_, 140 nM, final concentration) at room temperature for 20 min followed by the addition of [^3^H]DA (50 nM, final concentration) for an additional 8 min at room temperature. In parallel, nonspecific uptake was determined in the presence of 10 μM desipramine. Data are expressed as means ± S.E.M. from 4–9 independent experiments. * *p* < 0.05 compared to control value (in the absence of Tat, unpaired Student’s *t* test).

Our computational modeling predicted that mutation of Tyr467 to either Y467H or Y467 would considerably weaken the favorable NET-Tat binding (Fig 1). We validated the prediction by examining the specific [^3^H]DA uptake in WT hNET and the Tyr467 mutants in the presence or absence of 140 nM rTat_1-86_. As shown in Fig 3, the inhibitory effects of rTat_1-86_ on DA uptake via WT hNET, Y467H, and Y467F are presented as the ratio of [^3^H]DA uptake for each respective mutant in the presence of rTat_1-86_ compared to [^3^H]DA uptake in the absence of Tat. Addition of 140 nM rTat_1-86_ induced a 26.4% reduction of the specific [^3^H]DA uptake in WT hNET (with Tat, 2786 ± 410 DPM) relative to the control [without Tat, 3785 ± 427 DPM, *t*_(16)_ = 2.5; *p* < 0.05, unpaired Student’s *t* test], whereas Tat did not significantly alter the specific [^3^H]DA uptake in Y467H [in DPM: Tat (2848 ± 680) vs control (2784 ± 732)] or Y467F [in DPM: Tat (3416 ± 145) vs control (3568 ± 433)]. This finding indicates that the Y467 residue on hNET is crucial to HIV-1 Tat-mediated inhibition of DA uptake, suggesting that preventing HIV-1 Tat’s interaction with this residue may restore physiological DA levels in HIV-1 infected patients.

### Mutational effects of hNET on basal [^3^H]dopamine and [^3^H]MPP^+^ efflux

**Fig 4:**
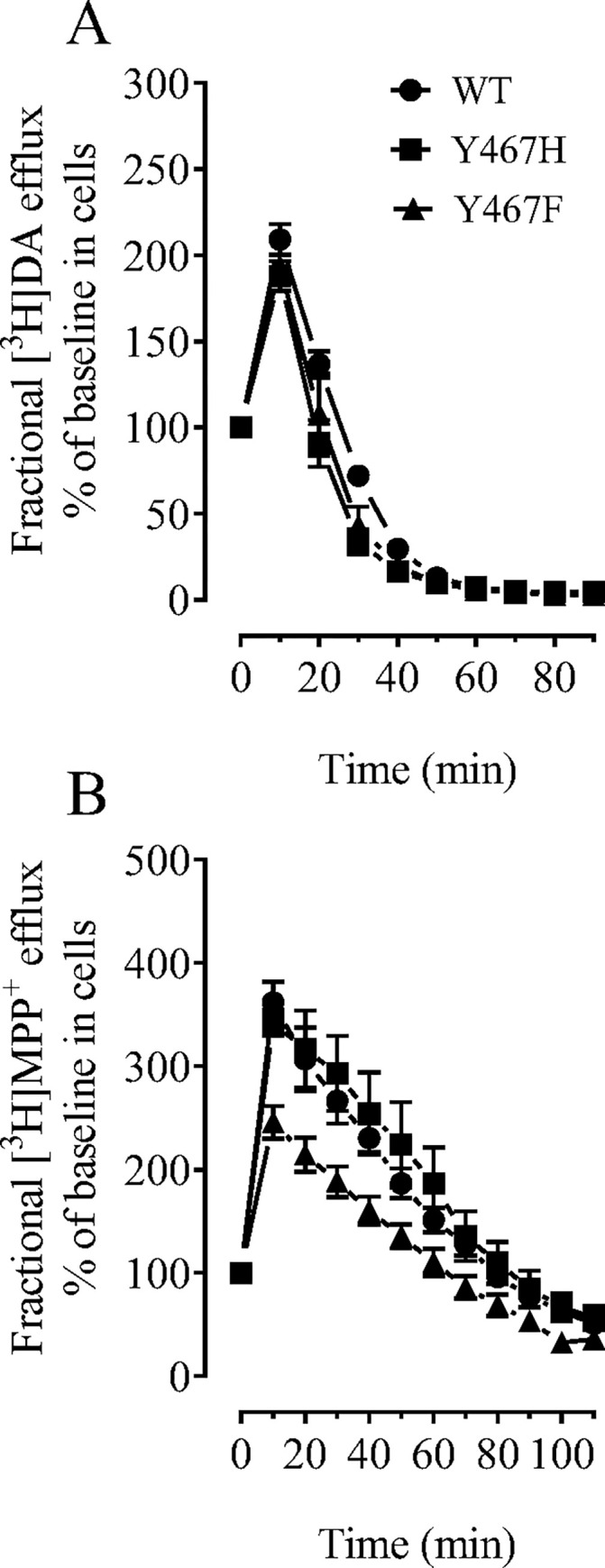
Mutational effect of hNET Tyr467 residue on functional efflux of DA or MPP^+^. CHO cells transfected with WT hNET or mutants were preincubated with assay buffer containing 50 nM [^3^H]DA for 20 min or 5 nM [^3^H]MPP^+^ for 30 min at room temperature. After incubation, cells were washed and incubated with fresh buffer at indicated time points. Subsequently, the buffer was removed, and radioactivity in the buffer and residual radioactivity in the cells was counted. Efflux fractions of [^3^H]DA or [^3^H]MPP^+^ in WT hNET or mutants were collected every 10 mins from 0 time point until 110 mins. Fractional efflux of [^3^H]DA or [^3^H]MPP^+^ are expressed as a percentage of baseline efflux at 0 time point at the start of the experiment. (A) [^3^H]DA efflux plotted as a percentage of baseline release after 20 minute preloading period. (B) [^3^H]MPP^+^ efflux plotted as a percentage of baseline release after 30-minute preloading period.

To further determine the effects of the mutations of Tyr467 residue on hNET on transporter conformational transitions, we examined the fractional efflux levels of [^3^H]DA and [^3^H]MPP^+^ in WT hNET and its mutants. As shown in Fig 4A, after 20-min preloading 50 nM [^3^H]DA, cells were washed and initial fractional efflux samples were immediately collected as 0 time point. While the efflux peak of [^3^H]DA was observed at 10 min in WT hNET and mutants, there was no difference in the efflux between WT hNET and mutants. With regards to the efflux of [^3^H]MPP^+^ (Fig 4B), a two-way ANOVA revealed significant main effects of mutation (F_(2, 6)_ = 5.4; *p* < 0.05), time (F_(10, 60)_ = 140.8; *p* < 0.001), and time × mutation (F_(20, 60)_ = 2.0, *p* < 0.05) for Y467F and Y467H when compared to WT hNET. Post-hoc analysis with a simple comparison for Y467F vs WT hNET revealed significant main effects of mutation (F_(1, 4)_ = 20.8, *p* < 0.05), time (F_(10, 40)_ = 110.5, *p* < 0.001), and time × mutation (F_(10, 40)_ = 3.527, *p* < 0.005) suggesting that overall efflux in Y467F was significantly reduced across 110 min. No difference in the efflux was observed between Y467H and WT hNET. These data support our findings that Y467F alters the conformation of the transporter, and additionally suggest that the Y467F mutation reduces the capability of the transporter to undergo efficient conformational transitions.

## Discussion

Our previous studies identified key residues in hDAT which are critical for Tat-induced inhibition of DAT and transporter conformational transitions [47–49, 64]. In the prefrontal cortex, in addition to hDAT, hNET also plays a critical role in reuptake of DA and NE. Since hDAT and hNET share a high degree of sequence homology and conformational similarities, it is likely that Tat-induced dysfunction of the DA system observed in HAND could be mediated by inhibition of both hDAT and hNET. Computational modeling and simulations predicted that the Tyr467 residue of the hNET, which mimics Tyr470 on the hDAT, is crucial for HIV-1 Tat induced inhibition of DA transport. Our major findings show that both Y467H and Y467F preserve the V_max_ of DA or NE uptake but attenuate Tat-induced inhibition of DA transport, which provide molecular insight into the modeled binding structure of the hNET-Tat complex.

Considering that Tat protein binds to the recognition residues in hDAT and hNET, this study evaluated the mutational effects of Tyr467 on basal hNET function as well as their ability to disrupt hNET-Tat interaction. Although the the Tyr467 residue of hNET is identical to Tyr470 in hDAT, where the mutants Y470H and Y470F decrease and retain DAT-mediated DA uptake, respectively [47, 48], both Y467H and Y467F retain the basal reuptake of DA or NE in hNET. However, both hDAT Tyr470 and hNET Tyr467 favorably interact with the positively charged amine group of M1 residue of Tat [62], which is evidenced by attenuating Tat-induced inhibition of DA uptake in both hDAT Tyr470 and hNET Tyr467. These findings suggest that HIV-1 Tat may inhibit both hDAT and hNET through a similar molecular mechanism. Indeed, consistent with the current results, our recent report demonstrates that DA uptake via mouseDAT or mouseNET is decreased in the prefrontal cortex of inducible Tat transgenic mice following 7- or 14-day administration of doxycycline, suggesting that the biological Tat expression disrupts both DAT and NET function [50]. Since Tat transgenic mice recapitulate the neuropathology and neurocognitive impairment observed in HIV infected individuals [65], understanding the molecular interactions of Tat protein and the recognition residues in hDAT or hNET may allow us to develop novel molecules for specifically blocking Tat binding on hDAT or hNET.

We have demonstrated that Tat-induced inhibition of hDAT is mediated by allosteric binding site(s) on hDAT, not by interaction with the DA binding site [62, 66, 67]. Of interest, this provides a molecular basis for using allosteric modulators to attenuate Tat binding to hDAT/hNET. Recent studies have reported that a unique quinazoline series of monoamine transporter partial antagonist [68] allosterically modulate transporter function [68, 69]. For example, our published work demonstrates that aallosteric modulator, SRI-30827, displays allosteric interaction with hDAT in binding and DA reuptake, and attenuates Tat-induced inhibition of hDAT function [70]. Interestingly, the SRI-30827 dissociates cocaine binding site on hDAT, which is attenuated in hDAT Y470F [70], suggesting that Tyr470 residue on hDAT is associated with SRI-30827-mediated allosteric activity. Based on the predictions of our computational modeling and pharmacological validation, we have identified several key recognition residues on hDAT for Tat binding, which display reduction, increase or unchanged in basal DA uptake via hDAT [47–49, 64]. The current study directly provides critical insights into developing new compounds that could block Tat binding on hNET but display a minimal effect on physiological DA/NE reuptake via hNET.

Our results show that Y467H but not Y467F mutant displays a decrease in the B_max_ of [^3^H]Nisoxetine binding without changing the B_max_ of [^3^H]WIN 35,428 binding, and potentiates the affinity of nisoxetine inhibiting [^3^H]DA uptake. This suggests that mutating Tyr467 to Tyr467His may alter the nisoxetine binding site in hNET, which could be induced by a transporter confirmation change. Our data is also consistent with a previous report showing that mutation of Tyr467 reduced the B_max_ of [^3^H]Nisoxetine and suggested it was due to decreased transporter expression [71]. However, our results from biotinylation and immunoblotting assays did not find a difference in total and surface expression of hNET between WT hNET and Y467H. Nisoxetine, a potent and selective hNET inhibitor, has been shown to induce inhibition through its interaction with the S2 binding site [72, 73], whereas WIN 35,428, a cocaine analog and a potent and selective hDAT inhibitor, induces inhibition via interactions with the S1 binding site [72, 74]. Both nisoxetine and WIN35,428 can interact with the respective binding sites on hNET [63]. The Tyr467 residue is located within a hydrophobic pocket of transmembrane helix 10 (TM10) of the hNET where it makes up a portion of the S2 binding pocket [71]. The S2 binding pocket is functionally responsible for allosterically triggering conformational changes from the occluded state to the inward-facing state, facilitating the release of ions and substrate, and the residues located in this pocket may take part in binding molecules before they reach the primary substrate binding site [74, 75]. One possibility is that Y467H mutant may induce a conformational change in the S2 binding pocket without interaction with the S1 site. Given that the dynamic transport process of hNET is similar to that observed in hDAT, determining the hNET mutation-induced conformational change is an interesting topic for further investigation.

The HIV-1 Tat protein has been shown to interact with DAT allosterically evidenced by altering zinc-regulation of DA uptake and WIN35,428 binding [66, 67]. Since there is not endogenous zinc binding site in hNET [76], the current study determined the underlying mechanism of hNET mutant-mediated attenuation of Tat-induced inhibition of [^3^H] DA uptake by assessing functional substrate efflux. We found that the DA efflux was not altered in Y467H and Y467F compared to WT hNET. However, the efflux of MPP^+^ was significantly reduced in Y467F, compared to WT hNET. Considering that the Y467 residue is critical for Tat interaction with hNET, these findings significantly highlight the impact of the Y467F mutant on the allosteric modulatory Tat interaction with hNET. While both DA and MPP^+^ are a substrate for NET [77, 78], MPP^+^ has less diffusive properties than DA in heterologous expression systems [79]. The current results demonstrate the differential influences of NET residues in the basal substrate efflux. Our previous study has demonstrated that Tat protein decreases DA efflux in WT hDAT [47]. Thus, further investigations including amphetamine-stimulated efflux and MTSET will be important to fully understand the mechanism of Tat-mediated changes in the conformational transition attributed to the identified residues in hNET.

In conclusion, we provide novel evidence that inhibition of DA uptake by HIV-1 Tat extends beyond the hDAT and affects DA uptake via the hNET as well. Attenuation of HIV-1 Tat-induced inhibition of hNET reuptake via mutation of hNET Tyr467 supports our computational modeling prediction of the HIV-1 Tat/NET interaction and suggests that this interaction may be similar to our previously reported HIV-1 Tat-hDAT interaction. Future studies will aim to investigate the contribution of multiple residues on hNET which have been predicted to be involved in the HIV-1 Tat-hNET interaction, which may provide molecular insight into the development of allosteric hDAT/hNET modulators that are capable of attenuating the inhibitory effects of HIV-1 Tat on DA reuptake.

## Supporting information

S1 File (DOCX)
