# Supplementary material for: Mutations of tyrosine 467 in the human norepinephrine transporter attenuate HIV-1 Tat-induced inhibition of dopamine transport while retaining physiological function
Source: PLoS One. 2022 Sep 28;17(9):e0275182. doi: 10.1371/journal.pone.0275182 (PMC9518868; doi:10.1371/journal.pone.0275182)
Supplement: S1 File — (DOCX) [file pone.0275182.s001.docx]

PONE-D-22-13812

Mutations of tyrosine 467 in the human norepinephrine transporter attenuate HIV-1 Tat-induced inhibition of dopamine transport while retaining physiological function

Matthew J. Strauss^1^, Katherine D. Porter^1^, Pamela M. Quizon^1^, Sarah E. Davis^1^, Steven Lin^1^, Yaxia Yuan^2,3^, Gustavo A. Martinez-Muniz^1^, Wei-Lun Sun^4^, Chang-Guo Zhan^2,3^, and Jun Zhu^1*^

^1^Department of Drug Discovery and Biomedical Sciences, College of Pharmacy, University of South Carolina, Columbia, SC

^2^Molecular Modeling and Biopharmaceutical Center, University of Kentucky, Lexington, KY

^3^Department of Pharmaceutical Sciences, College of Pharmacy, University of Kentucky, Lexington, KY

^4^Department of Psychological Science, University of North Georgia, Dahlonega, GA.

*Corresponding Author:

Jun Zhu, MD., PhD.

Department of Drug Discovery and Biomedical Sciences

College of Pharmacy, University of South Carolina

715 Sumter St, Columbia, SC 29208

E-mail: zhuj@cop.sc.edu (JZ)

Supporting inforamtion

S1 Fig.


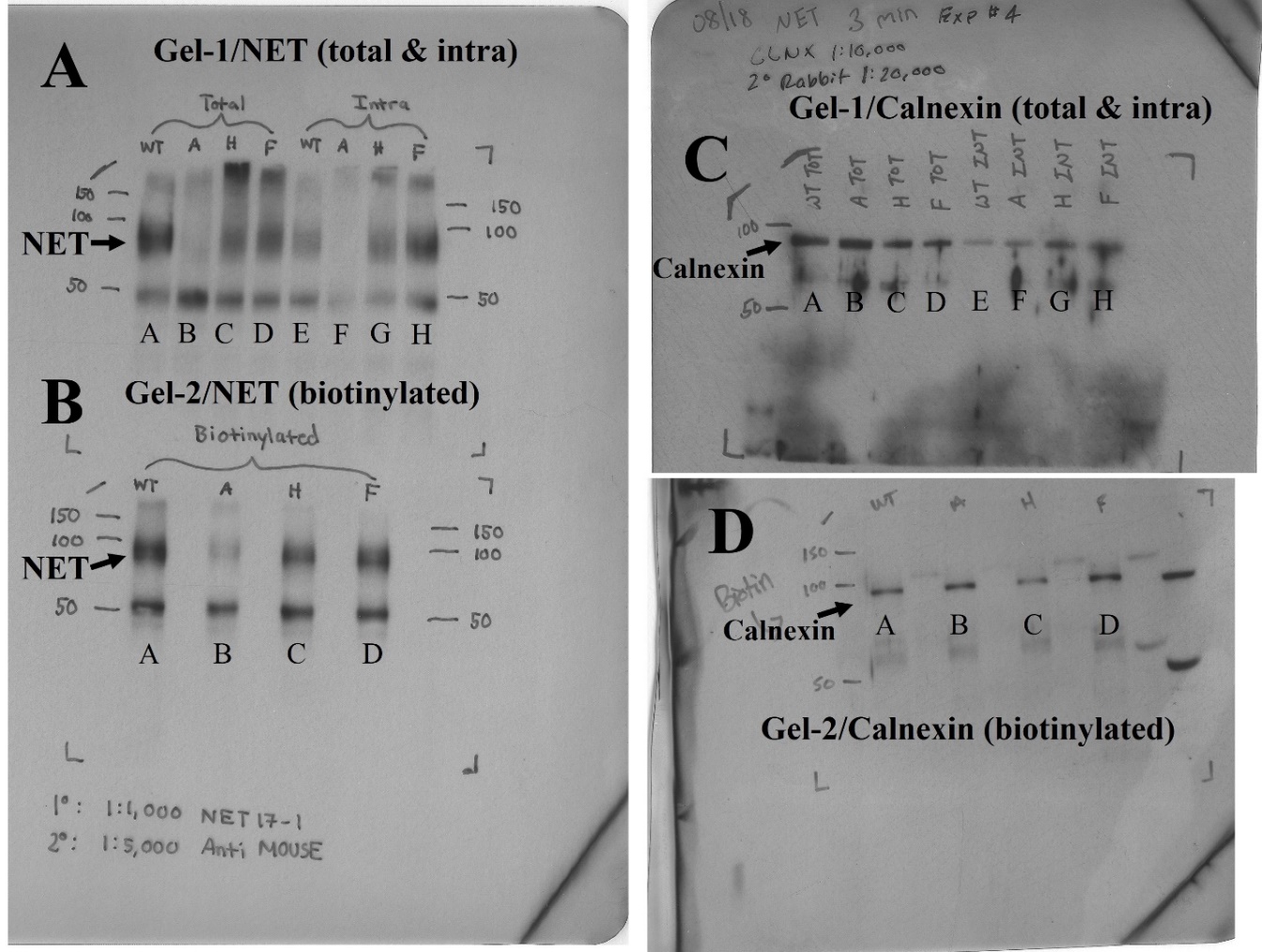


**This is the S1 Fig Title. Representative immunoblots for total, intracellular, and biotinylated NET (A and B) and Calnexin (C and D) in WT, Y467H, and Y467F mutant hNET expressing PC-12 cells.**

**This is the S1 Fig legend.** The total and intracellular fractions were prepared and loaded as described in the methods section under the biotinylation and western blot heading. The membrane was first probed using the 17-1 anti-NET antibody (A-B), then stripped using RestoreTM Western Blot Stripping Buffer (ThermoFisher, cat# 21059), and re-probed with the H70 anti-calnexin antibody (C-D) to monitor protein loading between all lanes. Lanes A, C, and D from panel A are shown in Figure 2A as total DAT fraction, and lanes E, G, and H from panel A are shown in Figure 2A as intracellular NET fraction. Lanes A, C, and D from panel B are shown as biotinylated fraction for NET in Figure 2A. The loading control for each fraction is shown in Figure 2A. The total is represented by lanes A, C, and D from panel C and the intracellular is represented by lanes E, G, and H from the same panel. The biotinylated calnexin fraction is shown from lanes A, C, and D from panel D.
